# Effects of Hot Air Drying on Drying Kinetics and Anthocyanin Degradation of Blood-Flesh Peach

**DOI:** 10.3390/foods11111596

**Published:** 2022-05-28

**Authors:** Si Tan, Yiwen Miao, Chongbing Zhou, Yuping Luo, Zhiru Lin, Ruobing Xie, Wenfeng Li

**Affiliations:** 1 School of Advanced Agriculture and Bioengineering, Yangtze Normal University, Chongqing 408100, China; mywfood@email.swu.edu.cn (Y.M.); 202009561114@stu.yznu.edu.cn (C.Z.); 201909561203@stu.yznu.edu.cn (Y.L.); 202009561117@stu.yznu.edu.cn (Z.L.); 202009561217@stu.yznu.edu.cn (R.X.); 20170152@yznu.edu.cn (W.L.); 2 College of Food Science, Southwest University, Chongqing 400715, China

**Keywords:** blood-flesh peach, hot air drying, drying characteristics, anthocyanins degradation

## Abstract

The purpose of this study was to explore the drying kinetics, effective moisture diffusivity, activation energy, color variation, and the thermal degradation properties of anthocyanins of blood-flesh peach under hot air drying for the first time. The results showed that the hot air-drying process of blood-flesh peach belongs to reduced-speed drying. The Page model could accurately predict the change of moisture ratio of blood-flesh peach. The effective moisture diffusivity during hot air drying of blood-flesh peach was in the range between 1.62 × 10^−10^ and 2.84 × 10^−10^ m^2^/s, and the activation energy was 25.90 kJ/mol. Fresh samples had the highest content (44.61 ± 4.76 mg/100 g) of total monomeric anthocyanins, and it decreased with the increase of drying temperature. Cyanidin-3-*O*-glucoside and delphinidin-3-*O*-galactoside were the main anthocyanins of blood-flesh peach as identified and quantified by UPLC-QqQ-MS. Interestingly, during the drying process, the content of cyanidin-3-*O*-glucoside increased at the beginning, and then decreased. However, the content of delphinidin-3-*O*-galactoside kept decreasing during the whole drying process. Considering the drying efficiency, fruit color and quality, 70 °C would be a suitable temperature for drying blood-flesh peach. This research will provide beneficial information for understanding the anthocyanin degradation of blood-flesh peach during drying, and guide the production of high-quality dried products.

## 1. Introduction

Peach (*Prunus persica* (L.) Batsch) fruit is one of the most important fruits in the world due to its delicious taste, fragrant flavor, and nutritional values. According to the color of the flesh, peach can be subdivided into white-flesh peach, yellow-flesh peach, and blood-flesh peach [1]. The blood-flesh peach with blood-red skin and flesh color is popular with consumers. In addition to the traditional nutrients such as sugar, acid, mineral, and vitamins, it has been reported that blood-flesh peach is also rich in phenolic compounds, especially anthocyanins [2]. Compared to other peach types, blood-flesh peach has much higher contents of anthocyanins and deserves to be particularly investigated.

Anthocyanins, a group of water-soluble pigments, are important secondary metabolites that contribute to the red, purple, orange, and blue colors of several fruits [3]. In addition to colorful characteristics, their antioxidant and anti-inflammatory activities have been extensively documented and supported in recent years [4]. The strong antioxidant property of anthocyanin contributes to its value in preventing cardiovascular diseases, neuronal diseases, cancer, and so on [4]. Anthocyanin has also been regarded as a potential natural colorant to replace synthetic colorants. Therefore, it is of importance both in the food industry and human health.

The seasonal availability and high susceptibility to quality deterioration after the harvest of blood-flesh peach limit their consumption [1]. Therefore, blood-flesh peach is usually processed as juice, can, dried slices, and so on. Drying is an effective method to extend the duration of long-term storage of fruits with high moisture [5]. Recently, several studies have been conducted to investigate the changes in nutrient composition of peaches under solar thermal drying [6], hot air drying (HAD), infrared drying, hot air-assisted radio frequency drying, and microwave-assisted hot air drying [7]. However, most of these studies focused on white-flesh peach or yellow-flesh peach. As far as we know, little information is available on the drying characteristics of blood-flesh peach. Furthermore, studies on the degradation curves of anthocyanins of blood-flesh peach during drying are lacking.

HAD is a harmless, non-toxic, and rapid drying process. It has been widely used to prolong the shelf-life during postharvest storage of several fruits [8]. It is well-known that HAD usually leads to changes in composition and contents of nutrients. For example, polyphenol contents and antioxidant activities of *Psidium guajava* fruits were drastically reduced after HAD [9]. Similarly, several studies indicated that thermal processing resulted in more than 50% loss in total phenolics in peaches [10]. On the other side, Arslan et al. reported that the antioxidant content of red bell-pepper was significantly increased by HAD [11,12]. Khattak et al. also found that HAD was the optimal drying process for acquiring mulberries with favorable texture and increased anti-oxidants [13]. Consequently, the effect of hot air drying on bioactive substances in fruits and vegetables varies with different subjects. Particularly, anthocyanin, as a heat-sensitive substance of water-soluble flavonoids, has very unstable chemical properties, and its stability is often destroyed by light and heat. However, the effects of drying temperature on anthocyanins degradation of blood-flesh peach during HAD have not been discussed.

The objective of this study was to explore the drying kinetics, determine the effective moisture diffusivity, activation energy (Ea) and color difference, and investigate the anthocyanin thermal degradation properties of blood-flesh peach under HAD at different temperatures (50, 60, and 70 °C). 

## 2. Materials and Methods

### 2.1. Chemicals and Standards

Formic acid was purchased from Sigma-Aldrich (St. Louis, MO, USA). Anthocyanin standards (purity ≥ 97%) were purchased from Shanghai yuan ye Bio-Technology Co., Ltd. (Shanghai, China). Acetonitrile for the ultra-high liquid phase was purchased from Shanghai Adamas Reagent Co., Ltd. (Shanghai, China). All the other reagents of analytical grade were purchased from Sichuan Chuandong Chemical Co., Ltd. (Sichuan, China).

### 2.2. Materials

Fresh blood-flesh peach was harvested from the garden of Xinyang, Henan Province, China. The blood-flesh peach pulp is blood-red, the texture is a little crisp, and the weight per fruit is around 200 g. To ensure convergence of internal moisture, blood-flesh peaches were placed in a refrigerator at 4 °C with constant temperature and humidity before the experiment. The initial moisture content of fresh blood-flesh peach was 91.56 ± 0.19 g H_2_O/g (dry basis, d. b.).

### 2.3. Drying Experiment

Before the drying experiment, the surface fluff of the blood-flesh peach needs to be cleaned. After removing the kernel, it was cut into 2 mm slices. The drying equipment was adjusted to the target parameters 30 min earlier to achieve steady-state conditions. The sample weight for each drying experiment was maintained at approximately 200 g. For HAD, an air blast dryer (101A, Shanghai Pudong Rong Feng Scientific Instrument Company, LTD., Shanghai, China) with constant temperature and humidity was used, where it was kept at 50, 60, or 70 °C and 70% relative humidity. The air velocity was 0.5 m/s. During the drying process, samples (10 pieces) were taken out from the dryer periodically to measure the weight. The time interval of weighting in the first 30 min of the drying process was 5 min, and it was increased to 15 min in the next 30 min, then increased to 30 min until the final moisture content was about 0.05 g/g (d.b.). The drying experiment was repeated three times. All samples were vacuum-sealed and stored at −20 °C until usage.

### 2.4. Drying Rate Curves

The moisture ratio (MR) and drying rate (DR) of peach slices were calculated according to Equations (1) and (2):

(1) $$\mathrm{MR}=\frac{M_{t}-M_{e}}{M_{0}-M_{e}}$$

(2) $$\mathrm{DR}=\frac{M_{t+dt}-M_{t}}{dt}$$

where *M_t_* is the moisture content at *t* time; *M*_0_ is the initial moisture content; *M*_e_ is the equilibrium moisture content; *M_t+dt_* is the moisture content at *t*+*dt* time; *dt* is the time elapsed for drying. All moisture content was expressed on a dry basis.

### 2.5. Drying Kinetics and Fitting Models

The HAD kinetic curves were fitted by five different MR models (Table 1), namely Lewis, Page, Modified midilli and other, Henderson and Pabis, and Wang and Singh models that were widely used in fruits and vegetables [14]. The drying models were screened and evaluated by chi-square value (*χ*^2^), the coefficient (*R*^2^), and the root-mean-square error (*RMSE*) values, where *χ*^2^, *R*^2^, and *RMSE* were calculated by Equations (3)–(5), respectively. A drying model is optimal for accurately predicting the variation of the moisture during drying when it has low *χ*^2^ and *RMSE* values and a high *R^2^* value.

(3) $$\chi^{2}=\frac{\sum_{i=1}^{N}{\left(MR_{exp,i}-MR_{per,i}\right)}^{2}}{N-n}$$

(4) $$R^{2}=1-\frac{\sum_{1}^{N}{\left(MR_{exp,i}-MR_{per,i}\right)}^{2}}{\sum_{1}^{N}{\left(\bar{MR_{exp}}-MR_{per,i}\right)}^{2}}$$

(5) $$RMSE={\left[\frac{1}{N}\sum_{i=1}^{N}{\left(MR_{pre,i}-MR_{EXP,i}\right)}^{2}\right]}^{\frac{1}{2}}$$

where *MR_exp_* is the experimental *MR*; *MR_pre_* is the predicted *MR*; *N* means the number of observations; *n* is the number of constants in the model.

### 2.6. Effective Moisture Diffusivities and Activation Energy

The effective moisture diffusivity (*D_eff_*) was calculated according to our previous report [15]. An Arrhenius-type relationship between *D_eff_* and the temperature was used to determine the activation energy (*E_a_*), which is expressed in Equations (6)–(8) [16].

(6) $$D_{eff}=D_{0}exp\left[\frac{-E_{a}}{R\left(T+273.15\right)}\right]$$

(7) $$\mathrm{In}D_{eff}=\mathrm{In}D_{0}-(\frac{E_{a}}{R}){\left(\frac{1}{T+273.15}\right)}^{\;}$$

(8) $$E_{a}=-a_{1}R$$

where *D_0_* is an integration constant, m^2^/s; *E_a_* is the activation energy, KJ/mol; *R* is the gas constant, 8.314 J/mol⋅K; *T* is the temperature, °C, and *a*_1_ is the slope of the line between ln (*D_eff_*) and 1/(*T* + 273.15).

### 2.7. Color Measurement 

The color of the fresh and dried blood-flesh peach slices was measured by a colorimeter (NR60CP, 3nh Technology, Shenzhen, China). Color was expressed in Hunter values (*L*, *a*, and *b*). The total color difference value (ΔE) was calculated according to Equation (9) to describe the color difference:

(9) $$\Delta E=\sqrt{{\left(L_{0}^{*}-L^{*}\right)}^{2}+{\left(a_{0}^{*}-a^{*}\right)}^{2}+{\left(b_{0}^{*}-b^{*}\right)}^{2}}$$

where *L*_0_, *a*_0_, and *b*_0_ are the values of fresh samples, respectively; *L*, *a*, and *b* are the measured values of dried samples.

### 2.8. Extracting

The anthocyanins of blood-flesh peach were extracted using the previous method with a slight modification [17]. One-gram fresh or dried peach samples were homogenized with dilute hydrochloric acid (pH = 2) solution (*w*/*v* = 1:10) for 1 min, and then extracted at 4 °C for 24 h. The solution was centrifuged at 10,000× *g* for 15 min and the supernatant was collected. All samples were extracted in triplicates.

### 2.9. Analysis of Total Monomeric Anthocyanins (ACY)

The content of ACY in blood-flesh peach was determined by the pH difference method [18]. Blood-flesh peach extract was added to pH 1.0 buffer solution and pH 4.5 buffer solution, respectively. The absorbances of two dilutions were analyzed by a microplate reader (PT3502PC, Beijing Potenov Technology Co., LTD, Beijing, China) at both 530 nm and 700 nm. The ACY was calculated as the cyanidin-3-*O*-glucoside (C3G) equivalent per 100 g fresh weight by the following Equation (10):

(10) $$ACY=\frac{A\times MW\times DF\times 10^{3}}{\varepsilon \times 1}$$

where *A* = [A_530_ − A_700_ (pH = 1.0)] − [A_530_ − A_700_ (pH = 4.5)]; *MW* is the molecular weight of C3G, 449.2 g⋅mol^−1^; *DF* is the dilution factor; *ε* is the molar absorptivity (26,900 L cm^−1^⋅mol^−1^) of C3G. All experiments were performed in triplicate.

### 2.10. Identification and Quantification of Anthocyanins by UPLC-QqQ-MS/MS

The anthocyanins of samples at five timepoints including the start, the end, the inflection point, the midpoint from start to the inflection point, and the midpoint from the inflection point to the end of the drying curves were identified and quantified by ultra-high-pressure liquid chromatography (UPLC, Agilent, Santa Clara, CA, USA), and triple quadruple mass spectrometry (6460 QqQ-MS/MS, Agilent, Santa Clara,, CA, USA) equipped with an electrospray ionization source (ESI). The ZORBAX SB-C18 column (100 mm × 2.1 mm i.d., 1.8 µm, Agilent, Santa Clara, CA, USA) was used to isolate the compounds. The mobile phase consisted of a gradient of 0.1% aqueous formic acid (A) and acetonitrile with 0.1% formic acid (B) at a flow rate of 0.2 mL/min. The gradient elution was set as follows: 0–6.8 min from 90% to 10% A, 6.8–7.0 min with 10% A, 7.0–7.5 min from 10% to 90% A, and 7.5–9.5 with 90% A. The sample injection volume was 5 μL, and the column temperature was 35 °C. The dynamic MRM (Multiple Reaction Monitoring) data were acquired, and the compounds were identified by comparing the dynamic MRM information with reference standards.

### 2.11. Statistical Analysis

All values in this study are presented as mean ± standard error. Statistically significant differences among the results were analyzed by one-way analysis of variance (ANOVA), followed by the Tukey test. The statistical analyses were performed using the IBM SPSS software (Version 20.0, SPSS Inc., Chicago, IL, USA). Microsoft Office excel (Version 2019, Microsoft Corp., Beijing, China) was used to prepare the figures. 

## 3. Results and Discussion

### 3.1. Drying Curves

It is well known that the drying temperature and time will affect the quality of dried fruits and vegetables. If the drying temperature is too low, the enzymatic browning of fruits and vegetables will increase and if the temperature is too high, most of the nutrients will be destructed. Therefore, it is of importance to select the most suitable drying temperature. 

**Figure 1:**
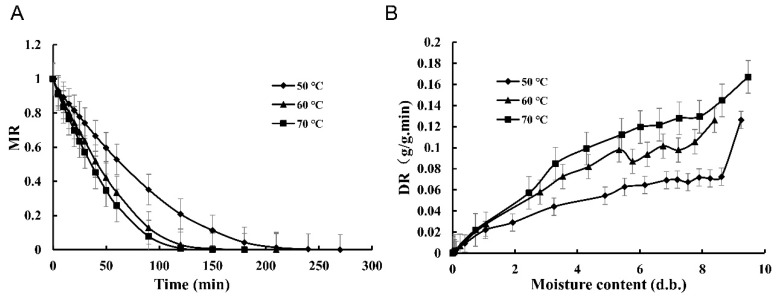
MR curves (**A**) and drying rate versus moisture content curves (**B**) of blood-flesh peach at different drying temperatures.

In this study, the drying characteristics of blood-flesh peach at different drying temperatures (50, 60, and 70 °C) during HAD were analyzed. As shown in Figure 1A, the MR curve of blood-flesh peach followed a decrease rate process, and the increase in temperature accelerated the drying process. When the drying temperature increased from 50 °C to 70 °C, the drying time was reduced from 270 to 180 min, indicating the mass transfer rate at 50 °C was lower than that at 70 °C. This was possibly attributed to the high moisture diffusion capacity at high temperature since the increased drying temperature could accelerate the activity of water molecules [15]. Similar observations were reported during HAD of mango slices [19] and *Boletus edulis* [10].

Figure 1B showed the DR curves of blood-flesh peach at different temperatures (50, 60, and 70 °C). It further indicated that the HAD process of blood-flesh peach belongs to reduced-speed drying. The initial DR at 70 °C (0.17 g/g min d.b.) is greater than the initial DR at 50 °C (0.13 g/g min d.b.). As it can be seen from Figure 1B, with the decrease in the drying temperature, the DR is also reduced, suggesting that elevated temperature can improve the drying rate of blood-flesh peach. This agrees with the previous study on the gas impingement jet drying of onions. Except for the temperature, the air velocity can also influence the drying rate [16]. In our study, fixed low air velocity was used. To obtain the optimal drying rate, advanced drying techniques such as air jet impingement drying, and heat pump drying with controlled air temperature and high air velocity would be good choices. 

### 3.2. Modeling the HAD Kinetics

A suitable mathematical modeling under different conditions is of importance to guide the actual drying process of fruits and vegetables. Five classic drying models were selected to fit the experimental data of blood-flesh peach. As summarized in Table 2, the Wang and Singh model and Page model showed high *R*^2^ (0.9945–0.9986), low *RMSE* (0.013670–0.026120), and *χ*^2^ (0.000531–0.000347) values for all drying conditions. Therefore, it was preliminarily determined that these two models might be suitable for predicting the HAD process of blood-flesh peach.

To select the most suitable model, further regression about the drying temperatures on the constant and coefficients were analyzed. According to the multiple regression analysis, the regression equation of the Page model equation can be expressed as $\mathrm{MR}=\exp\left[\left(-0.0002T+0.0052\right)t^{-0.0005T^{2}+0.0592T-0.5387}\right]$. The regression equation of the Wang and Singh model equation can be expressed as $\mathrm{MR}=1+(-0.0003T+0.007)t+(0.000002T-0.00007)t^{2}$, where *T* is air temperature, °C; *t* is the drying time, min.

**Figure 2:**
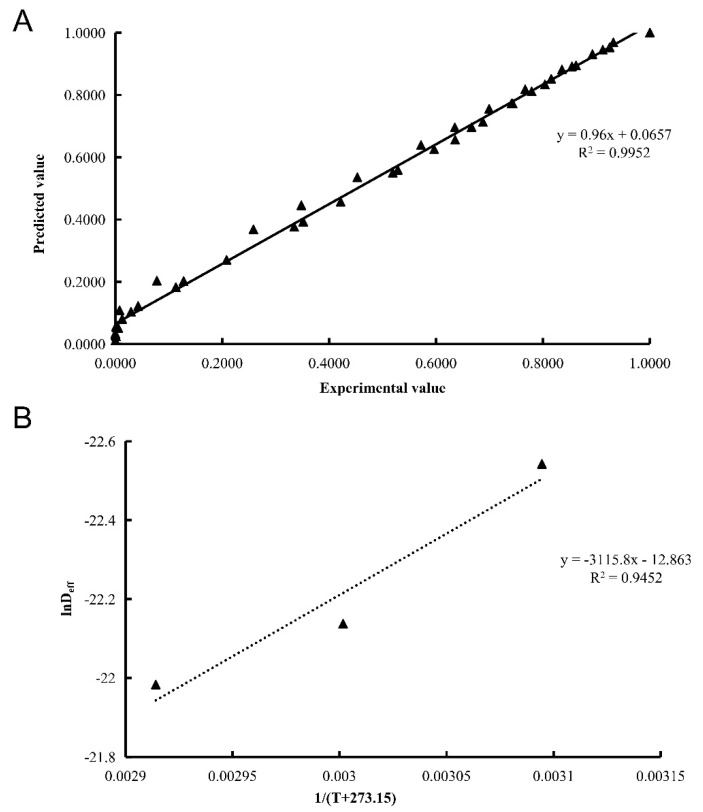
Comparison between experimental *MR* and predicted *MR* values of the Page model at different conditions (**A**). Arrhenius-type relationship between *D_eff_* and temperature (**B**).

Moreover, those two models were further validated by comparing the experimental values from all the tests with the predicted values of the models. As shown in Figure 2A, the predicted values of the Page model were consistent with the experimental values (*R*² = 0.9952). However, the correlation coefficient for Wang and Singh model was very low (*R*² = 0.4682). Therefore, the Page model was considered as the most suitable model to predict the moisture content of blood-flesh peach during HAD under the selected experimental conditions. Similarly, the Page model has been successfully applied in drying various agricultural products [20].

### 3.3. D_eff_ and E_a_

The *D_eff_* values of blood-flesh peach are shown in Table 2. It varied from 1.62 × 10^−10^ to 2.84 × 10^−10^ m^2^/s, and it was positively correlated with drying temperatures. This was mainly due to that increased temperature resulted in higher water diffusivity within the fruit, and a greater evaporation. Moisture distribution of samples, drying temperature, shrinkage degree of raw materials, and drying environment will affect the *D_eff_* of agricultural products [21]. Similar results were obtained for American ginseng root (*Panax quinquefolium*) (1.16 × 10^−10^ to 2.89 × 10^−10^ m^2^/s) by convective HAD [22].

The values of In*D*_eff_ versus the reciprocal of the temperature (1/(T + 273.15) were plotted in Figure 2B. The *E_a_* of blood-flesh peach was 25.90 kJ/mol, indicating that the energy required to dry 1 mol of water of blood-flesh peach is 25.90 kJ. Our result was similar to the result of yam (*Dioscorea hispida*) (23.53 kJ/mol) dried by a forced convective hot-air drier [23], lower than that of cucumber pericarp (35.03 and 35.82 kJ/mol) [24], but larger than that of blanch-assisted water yam (*Dioscorea alata*) (15.5 kJ/mol) [25]. Previous studies have proved that most agricultural products have different *E_a_* values in different drying processes, mainly in the range of 14.42 to 43.26 kJ/mol [5], and the variability might be caused by differences in raw materials, maturation status, tissue structure, specific surface area, and so on [16].

The energy consumption is also an important factor that needs to be considered in the industrial processing. The electricity energy cost per hour of HAD increased with the increase of drying temperatures (from approximate 1.6 Kw·h to 2.30 kW·h). However, a high drying temperature resulted in a short drying time (from 270 min to 180 min). Therefore, in our study, the total energy consumption at 70 °C was the lowest, but there was no significant difference among the three conditions.

### 3.4. Physical Characteristics

**Figure 3:**
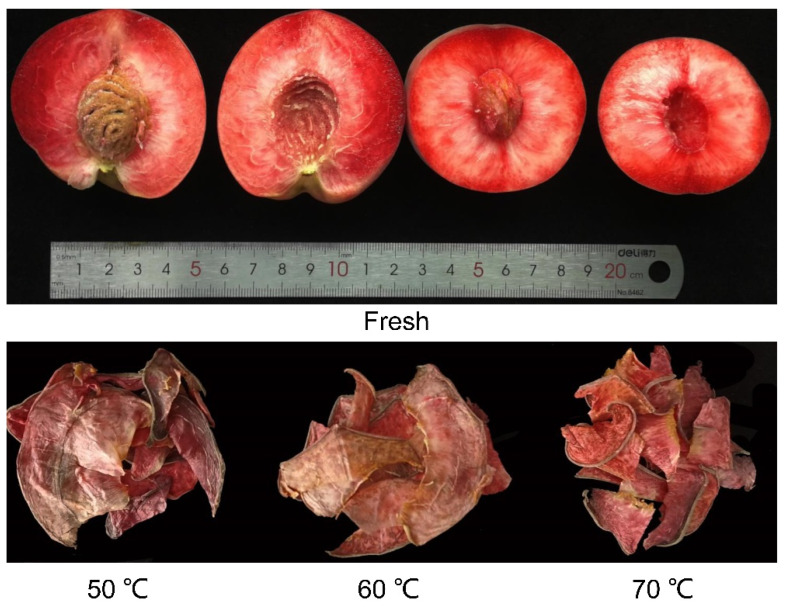
Fresh blood-flesh peach and dried samples under HAD at 50 °C, 60 °C, and 70 °C.

Color, as the primary sensory quality parameter, is an essential index for consumers to evaluate and purchase. The picture of fresh and dried samples is shown in Figure 3, and the values of *L* (brightness), *a* (red-green), and *b* (yellow-blue) are given in Table 2. The blood-flesh peach pulp was bright red and drying resulted in varying degrees of browning. The *L*, *a*, and *b* values of dried samples at different temperatures were significantly different from those of fresh samples. The *L* value decreased with the increase in drying temperature, but the difference among different dried samples was not significant. The *a* and *b* values representing redness and yellowness, respectively, of the dried samples were significantly lower than those of fresh samples. 

This could be due to the degradation of anthocyanins during HAD. It also can be seen that for the dried samples, the *a* and *b* values increased with the increase of temperature. This might be because drying at 50 °C needs longer time and then leads to more destruction of anthocyanins than that at 70 °C. On the contrary, the ∆E value was negatively correlated with temperature, and the sample dried at 50 °C had the highest total color difference value.

It can be concluded that the drying time and drying temperature had a certain effect on the color of blood-flesh peach. The observed difference in color between fresh samples and dried products may be caused by pigment degradation, enzymatic browning, and non-enzymatic reactions. Our result indicated that 50 °C caused the highest color variation. This might be because the enzymatic browning and Maillard reaction predominate over pigment destruction under this condition [16,26]. A similar result was reported in the study of change in color values of poppy petals during HAD [27]. However, the total color difference of Urmu mulberry increased with the drying treatment temperature [18]. The coefficient of color variation of blood-flesh peach with enzymatic browning, anthocyanin degradation, Maillard reaction, drying time, and drying temperatures need further investigation. 

As the dried peach slices are commonly consumed directly or smashed into powder as food ingredient, the texture of peach slices becomes an important quality affecting consumer acceptance. According to our sensory evaluation, peach slices dehydrated by HAD had significantly higher hardness than the fresh samples. Generally, drying at 70 °C would result in dense and tough products, whereas drying at lower temperature would produce less dense and loose slices. In addition, there was no obvious difference in crispness under different drying temperatures. Therefore, 70 °C could moderately increase the hardness and keep the crispness of peach slices. Normally, products with high hardness would not be preferred by consumers, but products with low hardness would easily be crushed during transportation. 

### 3.5. Anthocyanin Degradation

Anthocyanins are the primary pigments in blood-flesh peach. As shown in Figure 3A, the fresh samples had the highest content of total ACY (44.61 ± 4.76 mg/100 g), and HAD significantly decreased the ACY content. This was mainly due to the thermal degradation of anthocyanins under the condition of long-time heating [28]. It also can be seen that drying at 70 °C resulted in the most loss of anthocyanins. The ACY content in the dried samples showed a downward trend with the increase of temperature even though there was no significant difference. Similar results were reported in the study of the thermal stability of anthocyanins in blackberry juice [29]. 

To further understand the anthocyanin profile during HAD, UPLC-QqQ-MS/MS was used to identify and quantify the anthocyanins of blood-flesh peach. Two anthocyanins, named cyanidin-3-*O*-glucoside (C3G) and delphinidin-3-*O*-galactoside (D3G) were identified by comparing retention time and mass-to-charge ratio with standard substances. The content of C3G in blood-flesh peach is much higher than that of D3G (Figure 3B,C). This result was consistent with the literature that C3G accounts for most of the anthocyanins in peach [30].

**Figure 4:**
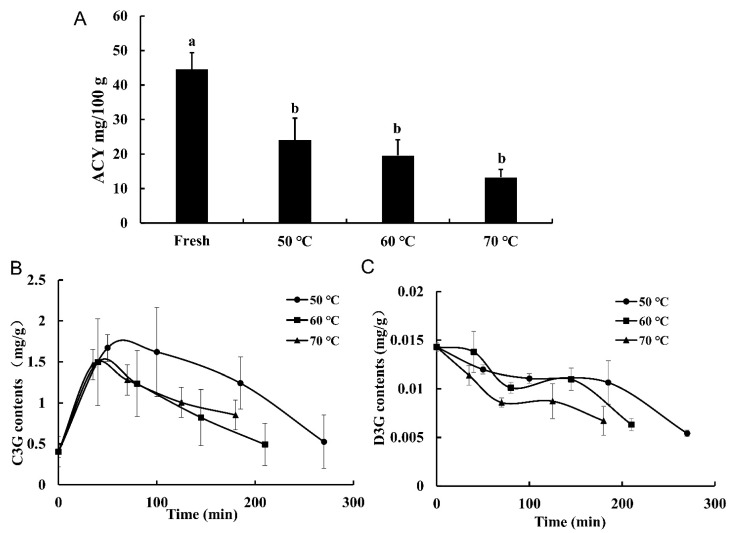
Contents of ACY in the fresh and dried blood-flesh peach (**A**). Degradation curves of cyanidin-3-glucoside (C3G) (**B**) and delphinidin-3-galactoside (D3G) (**C**) of blood-flesh peach during HAD. Values were presented as mean ± sd. Different letters mean significant difference as determined by Tukey’s post hoc test (*p* < 0.05).

C3G, as a natural red pigment, is consistent with the color presented by the blood-flesh peach, and it is vulnerable to temperature [31]. As shown in Figure 4B, the content of C3G in blood-flesh peach during drying showed a changing trend that increased firstly and then decreased gradually. No matter the drying temperature, the C3G content was the highest at the beginning of the drying process, even higher than the fresh samples. This phenomenon might be due to the accelerated thermal movement of anthocyanin molecules in high-temperature drying, which leads to an increase in the extraction rate of C3G [32]. However, with the extension of heating time, the structure of anthocyanin may be destroyed, resulting in the degradation of anthocyanin substances and a decrease in the content [33]. In addition, from the perspective of the action mechanism of enzymes, previous studies have suggested that the increase in anthocyanin content during the drying process of fruits and vegetables may be due to the increase of the activity of phenylalanine ammoniac lyase (PAL) and chalcone synthetase (CHS) with the increase of the internal temperature of materials. However, when the drying time was prolonged (60–120 min) or the temperature was increased, the activities of PAL and CHS were affected, resulting in no conversion of anthocyanins [34,35]. This result was consistent with the studies on the effects of temperature on anthocyanins of strawberry [36], and juice of “Merlot” and “Ruby” Grapes (*Vitis vinifera*) [37].

According to Figure 4C, the degradation mode of D3G was different from that of C3G, and showed a gradual decrease during the whole drying process. D3G is one of the main anthocyanins in blackcurrant, and it decreased with the increase in drying temperature [38]. Our study also showed that the contents of C3G and D3G in the sample dried at 50 °C were the highest during drying, and the temperature would affect the stability of C3G and D3G. However, in terms of the final contents of C3G and D3G, there was no significant difference among the samples dried by different temperatures. These results indicated that both the temperature and the drying time would affect the degradation of anthocyanins. Similar results have been reported previously [18,39].

## 4. Conclusions

In this study, we investigated the drying kinetics and anthocyanin degradation characteristics of blood-flesh peach under HAD. The HAD process of blood-flesh peach belongs to the reduced-speed drying, and the MR change pattern of blood-flesh peach under HAD conditions can be predicted by the Page model. The *D_eff_* coefficient of blood-flesh peach was in the range between 1.62 × 10^−10^ and 2.84 × 10^−10^ m^2^/s, which increased with temperature and was maximum at 70 °C. The *E_a_* of blood-flesh peach dried by HAD was 25.90 kJ/mol. Considering the drying efficiency, color, and quality of dried fruits, 70 °C was the most suitable HAD temperature for blood-flesh peach in our study. This finding can provide a guideline for improving the quality by optimizing the drying technology of blood-flesh peach. The next step of research could be investigating the role of PAL and CHS in the thermal degradation process of anthocyanin, to explore its mechanism of action, and to optimize the drying conditions.

## Figures and Tables

**Table 1:** Coefficients of different drying models for HAD of blood-flesh peach under different temperatures.

No.	Model	Formula	Parameter	Temperature/°C		
				50	60	70
1	Lewis	exp(−kt)	*k*	0.011940	0.017852	0.020864
			*R* ^2^	0.9843	0.9813	0.9847
			*χ* ^2^	0.001801	0.002079	0.001637
			*RMSE*	0.041167	0.044045	0.038988
2	Page	exp(−kt^n^)	*k*	0.004794	0.006266	0.008711
			*n*	1.212	1.272	1.236
			*R* ^2^	0.9964	0.9974	0.9975
			*χ* ^2^	0.000531	0.000379	0.000347
			*RMSE*	0.021652	0.018130	0.017256
3	Modified midilli and other	aexp(−kt) + b	a	1.133	1.111	1.102
			*k*	0.009641	0.016108	0.018818
			*b*	−0.124	−0.081	−0.077
			*R* ^2^	0.9968	0.9939	0.9952
			*χ* ^2^	0.000534	0.001048	0.000798
			*RMSE*	0.020202	0.027725	0.023872
4	Henderson and Pabis	aexp(−kt)	a	1.035	1.048	1.041
			*k*	0.012550	0.019076	0.022071
			*R* ^2^	0.9878	0.9869	0.9888
			*χ* ^2^	0.001623	0.001743	0.001416
			*RMSE*	0.037838	0.038869	0.034836
5	Wang and Singh	1 + at + bt^2^	a	−0.008939	−0.013199	−0.015440
			b	0.000020	0.000041	0.000056
			*R* ^2^	0.9986	0.9951	0.9945
			*χ* ^2^	0.000212	0.000737	0.000796
			*RMSE*	0.013670	0.025282	0.026120

**Table 2:** The effective moisture diffusivity and color values of blood-flesh peach at different temperatures. Values were presented as mean ± sd. Different letters in each line mean significant difference as determined by Tukey’s post hoc test (*p* < 0.05).

Temperature/°C	*D_eff_* (m^2^/s)	*L*	*a*	*b*	ΔE
Fresh	—	44.77 ± 1.81 b	24.32 ± 1.67 a	10.58 ± 1.33 a	-
50	1.62 × 10^−10^	52.27 ± 3.13 a	10.80 ± 2.27 b	3.92 ± 1.05 c	17.01 ± 0.97 a
60	2.43 × 10^−10^	51.97 ± 1.78 a	11.92 ± 2.91 b	6.17 ± 1.53 b	15.02 ± 1.71 a
70	2.84 × 10^−10^	49.54 ± 1.36 a	13.74 ± 2.40 b	6.26 ± 0.69 b	12.41 ± 0.07 b

## Data Availability

The data used in this study are available in this article.
